# Triptolide clears *Staphylococcus aureus* infection by targeting XIAP to induce host apoptosis while maintaining gut microbiota homeostasis

**DOI:** 10.3389/fphar.2026.1834558

**Published:** 2026-05-26

**Authors:** Xinli Qiu, Lihua Qiang, Yiru Wang, Bingxi Li, Zehui Lei, Jing Wang

**Affiliations:** 1 Key Laboratory of Pathogen Microbiology and Immunology, Institute of Microbiology, Chinese Academy of Sciences, Beijing, China; 2 State Key Laboratory of Medical Proteomics, National Center for Protein Sciences (Beijing), Academy of Military Medical Sciences, Beijing, China; 3 Medical School, University of Chinese Academy of Sciences, Beijing, China

**Keywords:** apoptosis, host-directed therapy, microbiota homeostasis, *Staphylococcus aureus*, triptolide, XIAP

## Abstract

**Background:**

*Staphylococcus aureus* (SA) remains a global health threat due to its increasing drug resistance and intracellular persistence, which compromise the conventional antibiotic efficacy. Host-directed therapy (HDT) has emerged as a promising alternative by modulating host immunity. With multi-targeting and immunomodulatory properties, traditional Chinese medicine (TCM) monomers represent ideal candidates for HDT. However, their ability to promote host immunity-mediated SA clearance remains largely unexplored.

**Methods:**

Forty-one TCM monomers potentially regulating host apoptosis, a core mechanism of the host innate immune defense against intracellular pathogens, were screened to identify a compound that promotes the clearance of intracellular SA and methicillin-resistant SA (MRSA). The mechanism was investigated in infected macrophages using transcriptomics, proteomics, molecular dynamics simulations, and biochemical assays. The physiological function of the TCM monomer was examined in infected mice through lung pathology and multi-omics analysis, including transcriptomics, proteomics, metagenomics, and metabolomics.

**Results:**

Triptolide was identified as a potent facilitator of host immunity-mediated intracellular clearance of SA and MRSA, without exerting direct bactericidal effects. Mechanistically, triptolide directly binds to the X-linked inhibitor of apoptosis protein (XIAP), disrupting its interaction with caspases to relieve their inhibition and thereby induce apoptosis. Furthermore, in murine infection models, triptolide treatment reduced bacterial loads, alleviated inflammation, and induced macrophage apoptosis in lungs, concurrently maintaining microbiota homeostasis and improving metabolic function.

**Conclusion:**

This study establishes a proof of concept for triptolide as a HDT candidate against SA and MRSA infections, which not only enhances host apoptosis-mediated pathogen clearance but also maintains host microbiota and metabolic homeostasis.

## Graphical Abstract

**Figure:**
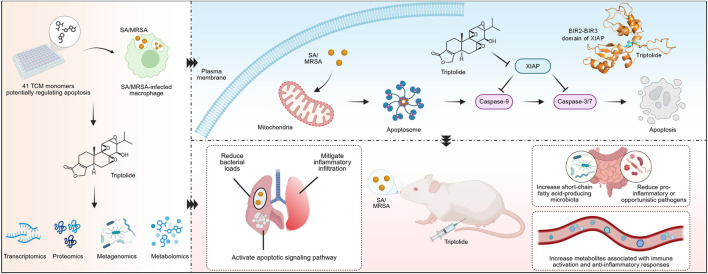

## 1 Introduction

*Staphylococcus aureus* (SA) constitutes a significant global health threat, responsible for a wide spectrum of hospital- and community-acquired infections. The current standard clinical management of SA infections primarily relies on antibiotics that directly target the pathogen. However, this approach poses a challenge of driving the emergence of drug-resistant strains, most notably exemplified by methicillin-resistant SA (MRSA), an important pathogen on the World Health Organization (WHO) Bacterial Priority Pathogens List (WHO, 2024). Studies have indicated that the mortality rate of infections caused by MRSA can be twice as high as that caused by susceptible strains, highlighting its severe clinical threat (Lewis et al., 2018). Moreover, beyond causing acute infections as an extracellular pathogen, SA can also invade and persist inside cells to cause chronic diseases such as pneumonia, a mode of infection that can evade conventional antibiotics (Goormaghtigh and Van Bambeke, 2024). Thus, there is an urgent need for developing next-generation, non-antibiotic therapies capable of eliminating intracellular infections and overcoming drug resistance.

Host-directed therapy (HDT) emerges as a promising strategy for overcoming drug resistance and intracellular persistence in SA infection, functioning to enhance host immunity while mitigating excessive inflammation (Zheng et al., 2025). With the multi-targeting and immunomodulatory properties, traditional Chinese medicine (TCM) monomers represent ideal candidates for HDT-based approaches (Dai et al., 2025). However, current research on TCM monomers against SA remains predominantly focused on direct antibacterial mechanisms, including baicalein against Staphylococcal Enterotoxin A, biochanin A against α-hemolysin, isorhaponitin against Sortase A/B, nepetin against Caseinolytic Peptidase P, and quercetin against biofilm formation (Liang et al., 2025). In contrast, studies investigating TCM monomers as HDT are relatively limited. Existing evidence suggests that TCM-mediated HDT primarily mitigates inflammatory responses induced by SA through mechanisms such as inhibiting the ERK, JNK MAPK, and NF-κB pathways (Shi et al., 2020), alleviating Pam3CSK4-induced inflammation (Li et al., 2020), and activating nuclear factor erythroid 2-related factor 2-mediated antioxidant and anti-inflammatory pathways (Zhang et al., 2025). Nevertheless, how TCM monomers actively enhance host immunity to promote bacterial clearance, rather than merely suppressing inflammation, remains largely unexplored. Thus, a systematic understanding of the immunomodulatory mechanisms by which TCM monomers boost host immune defense against intracellular SA is urgently needed to advance the development of HDT-informed, TCM-based anti-infective strategies.

Apoptosis serves as a core mechanism of host innate immune defense against intracellular pathogens. It not only restricts intracellular pathogen replication and dissemination through forming apoptotic bodies to induce non-lytic cell death, but also exerts immunomodulatory effects by avoiding inflammation and presenting antigens to activate adaptive immunity (Soe et al., 2021). Based on these, we aimed to identify key TCM monomers that can enhance host apoptosis during SA infection and to elucidate their underlying mechanisms. Through a small-scale screen of TCM monomers with potential apoptotic regulatory effects, we identified triptolide as a compound that facilitates intracellular clearance of SA and MRSA. Mechanistic studies revealed that triptolide directly binds to the X-linked inhibitor of apoptosis protein (XIAP) to inhibit XIAP-caspase interaction, thereby releasing caspase inhibition and inducing apoptosis. Moreover, treatment with triptolide reduced bacterial loads, alleviated inflammation, and induced macrophage apoptosis in the lungs of mice infected with SA and MRSA. Concurrently, it maintained gut microbiota homeostasis, including an increase in short-chain fatty acid-producing microbiota and a reduction in pro-inflammatory or opportunistic pathogens, along with elevated levels of metabolites linked to immune activation and anti-inflammatory responses, indicating the establishment of a homeostatic immunity. Taken together, these findings suggest triptolide as a promising lead compound for the development of HDT against SA and MRSA infections.

## 2 Materials and methods

### 2.1 Bacterial strains, cell lines, and TCM monomers

SA (ATCC, 6538) and MRSA (ATCC, 43300) were kind gifts of J. Feng (Institute of Microbiology, Chinese Academy of Sciences, Beijing, China) and were grown in lysogeny broth medium at 37 °C. Immortalized bone marrow-derived macrophages (iBMDMs) from mice on the C57BL/6 genetic background were kindly provided by F. Shao (National Institute of Biological Sciences, Beijing, China) and were cultured in Dulbecco’s modified Eagle’s medium (DMEM) with 10% fetal bovine serum (FBS). Forty-one TCM monomers used in this study were selected from a traditional Chinese medicine library (Selleck, L8300) based on their potential to regulate apoptosis and autophagy (Supplementary Table S1).

### 2.2 Macrophage infection and colony-forming unit counting (CFU)

iBMDMs were seeded in 12-well plates at a density of 5 × 10^5^ cells per well. Before infection, SA and MRSA were grown to mid-logarithmic phase and then pelleted, washed, and resuspended in DMEM medium with 10% FBS. iBMDMs were infected with SA or MRSA at a multiplicity of infection (MOI) of 10 with the treatment of DMSO (Sigma-Aldrich, D2650; negative control), methicillin (MedChem Express, HY-B0974; positive control), or the indicated TCM monomers. For inhibiting apoptosis or antagonizing XIAP, cells were treated with 25 μM Q-VD-OPh (MedChemExpress, HY-12305) or 0.2 µM SM-164 (Selleck, S7089), respectively. After 2 h, extracellular unentered bacteria were removed by washing three times with 1 × PBS, and cells were incubated for an additional 10 h in fresh DMEM with 10% FBS, 10 μg/mL gentamicin (Aladdin, G100392), and the corresponding TCM monomers. Subsequently, total macrophages along with the culture media were collected and centrifuged at 2,000 rpm at 4 °C for 5 min. The pellet was then lysed with 0.05% sodium dodecyl sulfate (SDS). Intracellular bacterial CFUs were quantified by plating serial dilutions of the lysates on LB agar, and colonies were counted after 24 h.

### 2.3 Minimal inhibitory concentration (MIC) assay

The MICs of triptolide against SA and MRSA were determined using the broth microdilution method in accordance with Clinical and Laboratory Standards Institute (CLSI) guidelines, with methicillin included as the control. Briefly, bacterial suspensions were adjusted to 1.0 × 10^6^ CFU/mL. Serial dilutions of triptolide (0–15 μg/mL) were prepared in LB broth in a final volume of 200 μL per well. Then, 100 μL of the adjusted bacterial suspension was added to each well. After 18 h of static incubation at 37 °C, the MIC was recorded as the lowest concentration that completely inhibited visible bacterial growth.

### 2.4 Scanning electron microscopy (SEM) analysis

SA and MRSA cultures were treated with triptolide at a final concentration of 10 μM (3.6 μg/mL), with methicillin (2.1 μg/mL) serving as the control. Following 6 h of incubation at 37 °C with shaking at 180 rpm, cells were harvested by centrifugation at 4,000 rpm for 10 min and washed twice with sterile PBS (pH 7.4). Samples were then fixed overnight at 4 °C with 2.5% glutaraldehyde. After three washes with 1 × PBS (5 min each), cells were dehydrated using a graded ethanol series (50%, 70%, 85%, 95%, and 100%). The dehydrated samples were subjected to critical point drying using Leica EM CPD300 and coated with gold using Hitachi E-1045 Ion Sputter Coater. Morphological changes were observed using SEM (Hitachi U8010).

### 2.5 Apoptosis analysis by flow cytometry

iBMDMs were infected with SA or MRSA for 8 h as described above. Cells were then collected and stained using the Annexin V-PI Apoptosis Detection Kit (YEASEN, 40302ES60) according to the manufacturer’s instruction. Briefly, collected cells were resuspended in 100 μL of 1 × Binding Buffer, followed by the addition of 5 μL of Annexin V-FITC and 10 μL of PI Staining Solution. The mixture was incubated at room temperature in the dark for 10–15 min. Cells were then washed once with 1 × PBS and fixed with 200 μL of 4% paraformaldehyde for 20 min. Following a final wash and resuspension in 1 × PBS, apoptotic cells including Annexin V^+^/PI^−^ (early apoptosis) and Annexin V^+^/PI^+^ (late apoptosis) cells were quantitated with BD FACS Calibur by recording at least 10,000 events per sample. Results were analyzed by FlowJo 10.4 software.

### 2.6 Immunoblotting analysis

Cells were lysed on ice using Western and IP lysis buffer (Beyotime, P0013) supplemented with 1 mM phenylmethylsulfonyl fluoride (PMSF; Beyotime, ST506). Lysates were centrifuged at 12,000 rpm for 10 min at 4 °C, and the supernatants were collected. Protein concentrations were determined using a BCA assay (Beyotime, P0009). Proteins were separated by SDS-PAGE and transferred to polyvinylidene difluoride membranes (Millipore, SLGV33RB). The membranes were blocked with 5% skimmed milk powder in Tris-buffered saline containing 0.1% Tween-20 (TBST) for 1 h at room temperature, and then incubated overnight at 4 °C with primary antibodies against caspase-3 (1:1,000; Cell Signaling Technology, 9,662), caspase-9 (1:1,000; Cell Signaling Technology, 9,502), GAPDH (1:5000; YTHX biotech, ZB002), and His (1:5000; ZSGB-Bio, TA-02). After washing three times with TBST (10 min each), the membranes were incubated with HRP-conjugated secondary antibodies for 1 h at room temperature. Following three final washes with TBST, the membranes were developed by Immobilon Western Chemiluminescent HRP Substrate (Sigma Aldrich, WBKLS0500) and exposed to x-ray film (Fujifilm, XBT-1).

### 2.7 Bio-layer interferometry (BLI) assay

Recombinant Bcl2 protein (Solarbio, P10880), STAT1 protein (Sino Biological, S52-52H-100), XIAP protein (Sino Biological, 10606-H07E1). BLI assay was carried out at 30 °C using Octet Red96e (Sartorius) with PBS containing 0.02% tween-20 (PBST). XIAP, STAT1, and Bcl2 were immobilized by His-tag on BLI sensors Octet® Ni-NTA (NTA) Biosensors using a concentration of 100 μg/mL, and then incubated with 100, 33, 11, 3, and 1 μM of triptolide. The signal from a reference sensor without immobilized protein was subtracted to remove background noise and aspecific binding. Association and dissociation phases were 180 s each, and data were fitted globally with a 1:1 binding model in the Octet Analysis software (Sartorius).

### 2.8 Molecular docking and molecular dynamics simulation

The two-dimensional (2D) structure of triptolide was retrieved from the PubChem database (https://pubchem.ncbi.nlm.nih.gov/), and the three-dimensional (3D) crystal structure of XIAP was acquired from the Protein Data Bank (PDB; https://www.rcsb.org/). Both structures were saved in PDB format. For molecular docking, the protein structure was pre-processed using PyMOL2 to remove water molecules and small ligands. The processed data were then imported into AutoDockTools 1.1.2 to add hydrogens and convert the file to PDBQT format, defining the protein as the receptor. Molecular docking analysis was performed using AutoDock Vina 1.1.2 to investigate the binding interactions and activities between the target and the ligand.

Molecular dynamics simulations were performed with Amber 24 to evaluate protein-ligand binding stability. The system was prepared using the ff14SB and GAFF force fields, solvated in TIP3P water, and neutralized with ions. Following energy minimization and equilibration under NVT and NPT ensembles, a 100 ns production simulation was conducted at 300 K and 1 bar. Trajectory analysis included calculations of RMSD, Rg, SASA, RMSF, hydrogen bonds, and binding free energy estimation via MM/GBSA.

### 2.9 Blocking assay

Five μg of His-XIAP was incubated with 20 μL Ni-NTA Agarose (Qiagen, 30230) in 500 μL of the binding buffer (50 mM Tris, pH 7.5, 150 mM NaCl, 5 mM DTT, and 0.1% NP-40) supplemented with 1% protease inhibitors cocktail for 2 h at 4 °C. The beads were then washed three times with the binding buffer and further incubated with cell lysates in the presence of either 4 μg triptolide or SM-164 for 4 h at 4 °C. Subsequently, the beads were washed and the bound protein complexes were subjected to immunoblotting analysis.

### 2.10 Mouse infection

The C57BL/6N mice were purchased from Vital River and maintained under barrier conditions in a BSL-2 biohazard animal room (12 h light/dark cycle, 50% relative humidity, at 25 °C–27 °C). Mice aged 8 weeks and sex-matched were anesthetized via intraperitoneal injection of 1% sodium pentobarbital (50 mg/kg), and were then inoculated intranasally with SA or MRSA (5 × 10^7^ CFU/mL). After infection, the mice were randomly assigned to the following treatment groups: PBS, triptolide (0.1 mg/kg), or methicillin (200 mg/kg). Two days post-infection, mice were euthanized and lungs were collected for further analysis (van der Meer et al., 2016; Tomlinson et al., 2023).

For bacterial burden analysis, lungs were homogenized with a FastPrep-24 System (MP Biomedicals), and the homogenates were plated by serial dilution on LB agar plates for 24 h. For histopathology analysis, lungs were fixed in 10% formalin and embedded in paraffin for hematoxylin and eosin staining. Slides were scanned with Aperio CS2 (Leica Biosystems) and quantitation of the inflammation area in each tissue section was performed using ImageJ 1.50e with an IHC Toolbox plugin (National Institutes of Health; https://imagej.nih.gov/ij/). For multiplex immunofluorescence analysis, paraffin-embedded lung sections were deparaffinized, underwent antigen retrieval in sodium citrate (pH 6.0), permeabilized with 0.2% Triton X-100, and blocked with 2% goat serum. Sections were then incubated with primary antibodies against cleaved caspase-3 (1:400; Cell Signaling Technology, 9661S) and F4/80 (1:500; HUABIO, HA721745), followed by Alexa-conjugated secondary antibodies. Slides were scanned using Aperio Versa 200 (Leica Biosystems) and quantitation of the cleaved caspase-3-positive cells in each lung section was performed using Imaris 9.6 (Bitplane; https://imaris.oxinst.com/).

### 2.11 Multi-omics analysis

For transcriptomics analysis, total RNA was extracted from iBMDMs infected with SA and treated with DMSO or triptolide, and from lung macrophages of mice infected with SA or MRSA treated with PBS, triptolide, or methicillin according to the TRIzol® manual (Life Technologies, 15596-026). RNA sequencing was conducted using the Illumina Novaseq 6000 platform with paired-end 150-bp reads. The adaptors and low-quality bases, which were evaluated using FastQC (v0.11.3) software, were trimmed by Trimmomatic (v0.39) software with the following parameters: TRAILING:20, MINLEN:235, and CROP:235. Trimmed reads were then aligned to the mouse genome (GRCm39) using STAR (v2.4.2a) (Dobin et al., 2013). The genes differentially expressed between different experimental groups with |log_2_ (fold change)| > 1 and the *P*-value <0.05 were analyzed by DESeq2 (v1.30.1) in R (v4.0.2) (Love et al., 2014).

For proteomics analysis, iBMDMs infected with SA and treated with DMSO or triptolide, and lung macrophages of mice infected with SA or MRSA treated with PBS, triptolide, or methicillin were analyzed by SDS-PAGE. Bands were excised from the Coomassie brilliant blue staining gel, rinsed with 100 mM ammonium bicarbonate, dried, and reduced using 10 mM dithiothreitol (DTT) in 100 mM ammonium bicarbonate at 56 °C, and then alkylated with 55 mM iodoacetamide. Proteins were then digested with 12.5 ng/μL trypsin overnight at room temperature. After trypsinization, 0.5 μg peptide mixture resolved in buffer A [0.1% formic acid (FA)] was loaded onto a 1 cm self-packed trap column (150 μm inner diameter, ReproSil-Pur C18-AQ, 3 μm; Dr Maisch) and separated on a 150 μm inner diameter column with a length of 15 cm (ReproSil-Pur C18-AQ, 1.9 μm; Dr Maisch) over a 78-min gradient [buffer A, 0.1% FA in water; buffer B, 0.1% FA in ACN/water (80:20)] at a flow rate of 600 nL/min (0–8 min, 6%–12% buffer B; 8–58 min, 12%–30% buffer B; 58–70 min, 30%–40% buffer B; 70–71 min, 40%–95% buffer B; and 71–78 min, 95% buffer B). The Orbitrap Fusion mass spectrometer (Thermo Fisher Scientific) was operated in the positive-ion mode at an ion transfer tube temperature of 320 °C. The positive-ion spray voltage was 2.0 kV. The Orbitrap Fusion Lumos was set to the OT–IT mode. For a full mass spectrometry survey scan, the target value was 5 × 10^5^ and the scan ranged from 300 to 1,400 m/z at a resolution of 120,000 and a maximum injection time of 50 ms. For the MS2 scan, a duty cycle of 3 s was set with the top-speed mode. Only spectra with a charge state of 2–6 were selected for fragmentation by higher-energy collision dissociation with a normalized collision energy of 35%. The MS2 spectra were acquired in the ion trap in rapid mode with an AGC target of 5,000 and a maximum injection time was set to dynamic, and the dynamic exclusion was set to 20 s. The mass spectrometry raw files were processed using MaxQuant (v2.1.0.0) and searched against the *Mus musculus* (C57BL/6J) reference proteomes from UniProt database (https://www.uniprot.org/proteomes/UP000000589) by using the Andromeda search engine. False discovery rate (FDR) was set to 1% on peptide and protein levels with a minimum length of seven amino acids and was determined by searching a reverse database. Peptide identification was performed with an allowed initial precursor mass deviation up to 7 ppm and an allowed fragment mass deviation of 20 ppm. For all other search parameters, the default settings were used. The genes differentially expressed between different experimental groups with the |log_2_ (fold change)| > 1 and the *P*-value < 0.05 were analyzed by limma (v 3.46.0) in R (v4.0.2) (van Ooijen et al., 2018).

For metagenomic analysis, fecal specimens were collected from mice infected with SA or MRSA and treated with PBS, triptolide, or methicillin. Samples were collected in sterile tubes, immediately frozen, and stored at −80 °C until further analysis. Fecal DNA was extracted using a ONREW Magnetic Bead Method Fecal DNA Extraction Kit (ONREW, PDT302-02). Genomic DNA was randomly fragmented by Covaris to an average size of 300–350 bp. The fragments were treated with End Prep Enzyme Mix for end repairing, 5′ Phosphorylation and 3′ adenylation, to add adaptors to both ends. Each sample was then amplified by PCR for 8 cycles using P5 and P7 primers, with both primers carrying sequences which can anneal with flowcell to perform bridge PCR and P7 primer carrying a six-base index allowing for multiplexing. The PCR products were cleaned up and validated using an Agilent 2100 Bioanalyzer. The qualified libraries were sequenced with paired-end 150-bp reads on the DNBSEQ-T7 High-Throughput Genetic Sequencer. The quality statistics software Cutadapt (v1.9.1) was used to remove primers and low-quality sequences. The BWA software (v0.7.12) was used to compare with the host genome and filter out the reads that might be of host origin. The remaining sequences were aligned to the NR database using diamond (v2.1.10.164) for taxonomic annotation. The relative abundance of taxa at different taxonomic levels (phylum to species) was calculated by summing the abundances of annotated genes in each sample. Microbiome diversity analyses were performed using the R package vegan (v2.6.4). Alpha diversity analysis was performed using the Simpson index. Differential taxa and functional features between groups were identified using the Metastats method (Paulson et al., 2011).

For metabolomic analysis, blood samples were collected via retro-orbital bleeding into sterile microtubes. The serum was separated by centrifugation and immediately stored at −80 °C to preserve metabolite integrity. Thawed serum samples were analyzed using a Biocrates Absolute IDQ p180 kit (Biocrates Life Sciences AG, Innsbruck, Austria) for targeted metabolite quantification, following the manufacturer’s established protocols. LC-MS/MS analyses were performed using an UHPLC system (Vanquish, Thermo Fisher Scientific) with a Waters ACQUITY UPLC BEH Amide (2.1 mm × 50 mm, 1.7 μm) coupled to Orbitrap Exploris 120 mass spectrometer (Orbitrap MS, Thermo). The mobile phase consisted of 25 mmol/L ammonium acetate and 25 ammonia hydroxides in water (pH = 9.75) (A) and acetonitrile (B). The auto-sampler temperature was 4 °C, and the injection volume was 2 μL. The Orbitrap Exploris 120 mass spectrometer was used for its ability to acquire MS/MS spectra on information-dependent acquisition (IDA) mode in the control of the acquisition software (Xcalibur, Thermo). In this mode, the acquisition software continuously evaluates the full scan MS spectrum. The ESI source conditions were set as follows: sheath gas flow rate as 50 Arb, aux gas flow rate as 15 Arb, capillary temperature as 320 °C, full MS resolution as 60,000, MS/MS resolution as 15,000, collision energy as SNCE 20/30/40, spray voltage as 3.8 kV (positive) or −3.4 kV (negative), respectively. The raw data were converted to the mzXML format using ProteoWizard (v3.0.26042) (Chambers et al., 2012). The missing values were filled up by half of the minimum value. Data was scaled and logarithmically transformed to minimize the impact of both noise and high variance of the variables. PLS-DA (partial least-squares discriminant analysis) was carried out to visualize the distribution and the grouping of the samples by using the R package mixOmics (v 6.14.1). Furthermore, variable importance in the projection (VIP) of the first principal component in PLS-DA analysis was obtained to summarize the contribution of each metabolite. The metabolites with VIP > 1 and *P*-value < 0.05 (Student’s t-test) were considered as significantly changed metabolites.

For enrichment analysis, gene ontology (GO), Kyoto Encyclopedia of Genes and Genomes (KEGG), and Reactome enrichment analysis were carried out using the R package clusterProfiler (v3.6.0), and the enriched items with BH-adjusted *P*-value <0.05 were considered to be statistically significant. The single-sample GSVA score for a biological pathway was calculated using the “gsva” function in the R package GSVA (v1.38.2). The gene sets of biological pathways were obtained from Molecular Signatures Database (MSigDB) (https://www.gsea-msigdb.org/gsea/index.jsp). The visualization of differentially abundant microbes and pathways was performed using the R package pheatmap (v1.0.12.4).

### 2.12 Statistical analysis

Experimental data were statistically analyzed using GraphPad Prism 8.0. Statistical significance was assessed using one-way or two-way ANOVA according to the experimental design, with multiple comparisons conducted via Tukey or Dunnett post-hoc tests. *P* > 0.05, not significant (ns); *P < 0.05; **P < 0.01; ***P < 0.001; ****P < 0.0001. Data are presented as mean ± standard error of mean (s.e.m.) and log_2_-based transformation, according to data distribution. Unless otherwise indicated, at least three biological replicates were included in all experiments. Additional details about the statistical analysis of experiments and number of biological replicates (*n*) are indicated in the corresponding figure legends.

## 3 Results

### 3.1 Identification of triptolide as a compound facilitating the clearance of intracellular SA and MRSA

**FIGURE 1:**
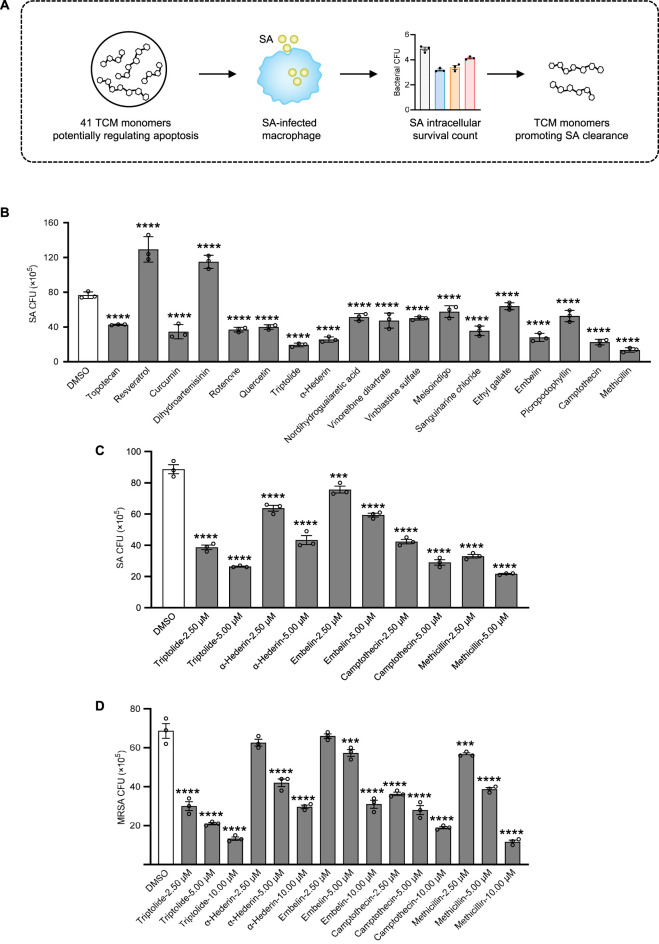
Triptolide promotes the clearance of intracellular SA and MRSA. **(A)** Schematic diagram of the procedure for screening TCM monomers that modulate intracellular SA clearance. **(B)** Representative results illustrating the effects of 15 TCM monomers that promoted intracellular SA clearance and 2 that inhibited it. DMSO and methicillin were used as the negative and positive controls, respectively. Intracellular survival analysis of SA **(C)** and MRSA **(D)** in iBMDMs treated with different concentrations of the designated TCM monomers. Data are shown as mean ± SEM [n = 3 in **(B–D)**]. ***0.0001 ≤ *P* < 0.001; ****P < 0.0001 [one-way ANOVA with Dunnett’s *post hoc* test for **(B–D)**].

Apoptosis is the critical host defense mechanism clearing intracellular pathogens (Hu et al., 2025). To identify TCM monomers that promote the clearance of intracellular SA, we firstly selected 41 candidates potentially regulating apoptosis from a traditional Chinese medicine library containing 1,933 compounds (Supplementary Table S1), and then subjected them to the SA intracellular survival assay (Figure 1A). The results revealed that 15 compounds enhanced intracellular SA clearance, while 2 compounds promoted bacterial intracellular persistence (Figure 1B). Among these 15 clearance-enhancing compounds, triptolide, α-hederin, embelin, and camptothecin exhibited the strongest efficacy (Figure 1B). Subsequently, we subjected these 4 compounds to a dose-response assay, reducing their concentrations from the initial 10.0 μM–5.0 μM and 2.5 μM. Notably, all tested compounds retained their SA clearance-promoting efficacy even at the lower doses, with triptolide exhibiting the most pronounced activity (Figure 1C). Furthermore, to elucidate whether these 4 TCM monomers could enhance the intracellular clearance of MRSA, a major drug-resistant SA strain, we established an MRSA-infected iBMDM cell model and administered graded concentrations of these compounds to the infected cells. Our findings demonstrated that almost all the monomers exerted effective MRSA clearance activity across all tested concentrations, with the only exceptions being α-hederin and embelin at the 2.5 μM dose (Figure 1D). Moreover, triptolide consistently exhibited the robust pathogen clearance efficacy (Figure 1D). Taken together, triptolide represents a critical TCM monomer that facilitates the intracellular clearance of both SA and MRSA.

### 3.2 Triptolide enhances macrophage apoptosis to clear SA and MRSA

Triptolide is an abietane-type diterpenoid isolated from *Tripterygium wilfordii* Hook. F., which exhibits broad-spectrum pharmacological activities against tumors, inflammatory disorders, and neurodegenerative diseases (Gao et al., 2021), but its regulatory functions and mechanisms in infections remain largely unexplored. Next, we investigated the mechanism by which triptolide facilitates the intracellular clearance of SA and MRSA. To begin with, we assessed whether triptolide possesses direct antibacterial activity. The MIC of triptolide against both SA and MRSA was greater than 15 μg/mL (approximately 41.62 μM), a concentration substantially higher than the effective dose (2.5 μM) used in SA/MRSA-infected macrophage models (Supplementary Figures S1A,B). Furthermore, SEM analysis revealed that triptolide treatment did not induce detectable morphological damage or cell wall disruption in either SA or MRSA, whereas the positive control antibiotic methicillin caused substantial damage to SA and moderate alteration to MRSA (Supplementary Figure S1C). Thus, these data suggest that triptolide promotes the intracellular clearance of SA and MRSA primarily by modulating host cellular functions, rather than through direct antibacterial activity.

**FIGURE 2:**
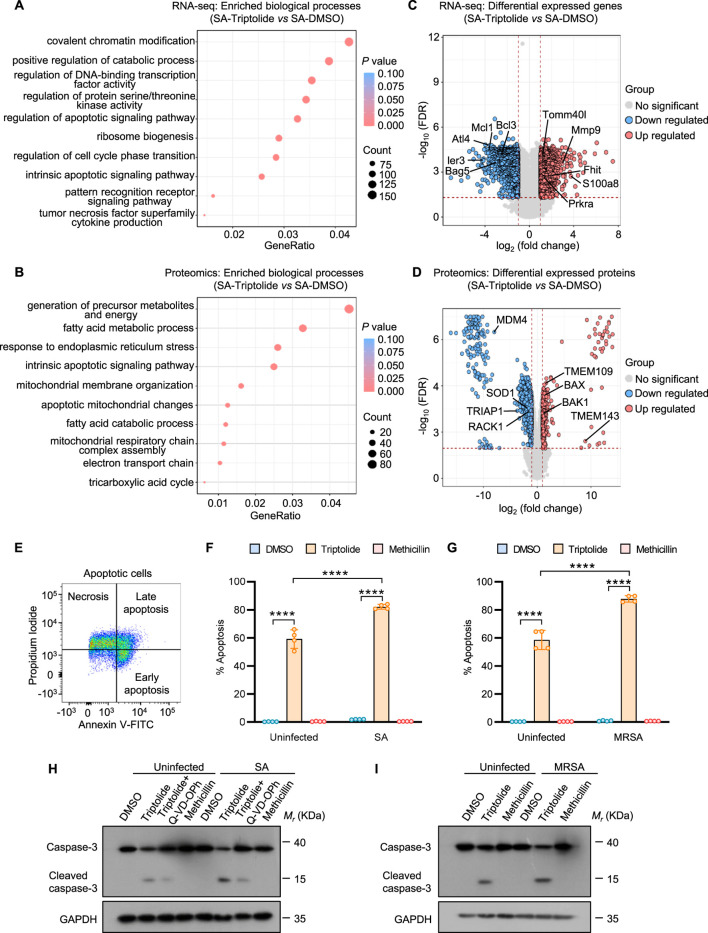
Triptolide triggers activation of the apoptotic signaling pathway. Gene Ontology (GO) enrichment analysis of differentially expressed genes **(A)** and proteins **(B)** in SA-infected iBMDMs treated with triptolide versus DMSO. Volcano maps depicting differentially expressed genes **(C)** and proteins **(D)** in SA-infected iBMDMs treated with triptolide versus DMSO. **(E)** Representative results of fluorescence-activated cell sorting (FACS)-based analysis for percentages of apoptotic cells. Quantification of apoptosis in SA **(F)**- and MRSA **(G)**-infected cells treated with DMSO, triptolide, or methicillin. Immunoblotting analysis of cleaved caspase-3 in SA **(H)**- and MRSA **(I)**-infected cells treated with DMSO, triptolide, or methicillin. Q-VD-OPh was used as the caspase inhibitor. Data are shown as mean ± SEM [n = 4 in **(F,G)**]. ****P < 0.0001 [two-way ANOVA with Tukey’s post-hoc test for **(F,G)**].

Next, we performed integrated transcriptomic and proteomic analyses to investigate how triptolide regulates host functions, as this dual approach enables a complementary view of both regulatory potential and functional effectors. Enrichment analysis identified a significant enrichment of biological processes associated with apoptosis regulation (Figures 2A,B). Moreover, differentially expressed genes (DEGs) analysis demonstrated marked upregulation of pro-apoptotic genes including *S100a8* and *Fhit* and concurrent downregulation of anti-apoptotic genes such as *Mcl1* and *Bcl3* (Figure 2C). Proteomic data corroborated these findings, revealing substantial elevation of pro-apoptotic proteins (e.g., BAX and BAK1) alongside a reduction in anti-apoptotic proteins (e.g., MDM4 and SOD1) (Figure 2D). Collectively, these data indicate that triptolide promotes the activation of the host apoptotic pathway. To further validate this pro-apoptotic effect of triptolide during SA and MRSA infections, we performed the Annexin V-FITC/PI dual staining, which indicates early apoptotic cells as Annexin V^+^PI^−^ and late apoptotic cells as Annexin V^+^PI^+^, respectively (Hasper et al., 2000). The results demonstrated that triptolide markedly increased the proportion of apoptotic cells during SA and MRSA infections (Figures 2E–G). Moreover, as the major executioner of apoptosis, caspase-3 is cleaved to generate active heterodimers that trigger downstream substrate proteolysis and apoptosis activation. We therefore examined the proteolytic cleavage of caspase-3 and found that triptolide substantially enhanced this process, while this effect was attenuated by Q-VD-OPH (Figures 2H,I), a broad-spectrum caspase inhibitor with potent antiapoptotic properties (Caserta et al., 2003). Finally, treatment with Q-VD-OPH significantly abrogated triptolide-facilitated intracellular clearance of SA and MRSA (Supplementary Figure S2). Taken together, these results demonstrate that triptolide enhances SA and MRSA clearance by promoting macrophage apoptosis.

### 3.3 Triptolide binds XIAP to disrupt caspase inhibition and thus induces apoptosis

**FIGURE 3:**
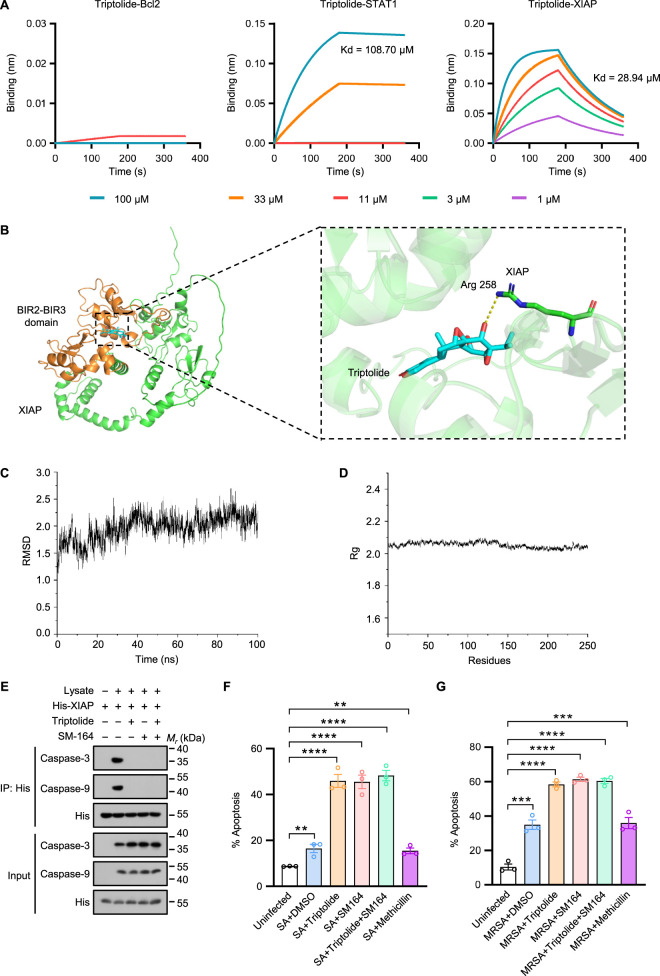
Triptolide induces apoptosis through targeting XIAP. **(A)** Measurement of triptolide binding affinity to Bcl2, STAT1, and XIAP by octet. **(B)** Molecular docking analysis between triptolide and XIAP. The BIR2 and BIR3 domains of XIAP are shown in orange. RMSD **(C)** and Rg **(D)** curves depicting the interaction between triptolide and XIAP. RMSD, root mean square deviation; Rg, radius of gyration. **(E)** Blocking assay to assess the effect of triptolide or SM-164 on XIAP-caspase-3/9 interaction. Quantification of apoptosis in SA **(F)**- and MRSA **(G)**-infected cells treated with DMSO, triptolide, SM-164, triptolide in combination with SM-164, or methicillin. Data are shown as mean ± SEM [n = 3 in **(F,G)**]. **0.001 ≤ *P* < 0.01; ***0.0001 ≤ *P* < 0.001; ****P < 0.0001 [one-way ANOVA with Dunnett’s *post hoc* test for **(F,G)**].

We next sought to identify the molecular target of triptolide that mediates its pro-apoptotic effect. Three candidate proteins, including B cell lymphoma 2 (Bcl2), signal transducer and activator of transcription 1 (STAT1), and XIAP, were initially screened using the Traditional Chinese Medicine Systems Pharmacology Database and Analysis Platform (TCMSP, https://www.tcmsp-e.com/tcmsp.php). To validate the direct binding affinity between triptolide and these candidates, octet assays were performed and revealed that triptolide bound specifically to XIAP, but not to Bcl2 or STAT1 (Figure 3A). XIAP is a well-established anti-apoptotic protein that functions by binding to and repressing caspase activity (Hanifeh and Ataei, 2022). To further characterize the binding interface between triptolide and XIAP, we performed molecular docking analysis and found that triptolide interacted with both the BIR2 and BIR3 domains of XIAP, with Arg258 identified as a key residue (Figure 3B). This binding mode was further validated by molecular dynamics simulations, which demonstrated the stable triptolide-XIAP (BIR2 and BIR3 domains) interaction as evidenced by low root mean square deviation (RMSD) (within 2 Å) and stable radius of gyration (Rg) fluctuations (Figures 3C,D), persistent hydrogen bonding (0–1 bonds), limited amino acid residue flexibility in binding regions, and favorable binding free energy (ΔG_binding = −149.92 ± 12.3909 kJ/mol) dominated by van der Waals and electrostatic interactions (Supplementary Figure S3). Given that the BIR2 and BIR3 domains of XIAP selectively target caspase-3/7 and caspase-9, respectively, to mediate anti-apoptotic signaling, we hypothesized that triptolide, as a dual-domain binding ligand, might serve as a potent XIAP antagonist.

To test the above hypothesis, we examined the interaction between XIAP and caspase 3/9 in the presence or absence of triptolide. Our data showed that triptolide treatment effectively abrogated the formation of XIAP-caspase-3/9 complexes, an effect comparable to that of SM-164 (Figure 3E), a well-characterized XIAP antagonist that targets both the BIR2 and BIR3 domains (Sun et al., 2007). Moreover, triptolide alone, SM-164 alone, or their combination showed similar efficacy in blocking these interactions (Figure 3E), supporting a shared mechanism of action. Subsequently, we further investigated the functional relevance of the triptolide-XIAP interaction during SA and MRSA infections. Triptolide treatment induced a level of apoptosis that was comparable to that induced by SM-164 alone or the combination of triptolide and SM-164 (Figures 3F,G). Consistently, triptolide exhibited a similar capacity to SM-164 (either as a single agent or in combination) in facilitating the intracellular clearance of SA and MRSA (Supplementary Figure S4). Collectively, these findings demonstrate that triptolide binds XIAP to relieve caspase inhibition and induce apoptosis, thereby promoting intracellular clearance of SA and MRSA.

### 3.4 Triptolide potentiates host apoptosis-mediated anti-SA/MRSA immunity *in vivo*

**FIGURE 4:**
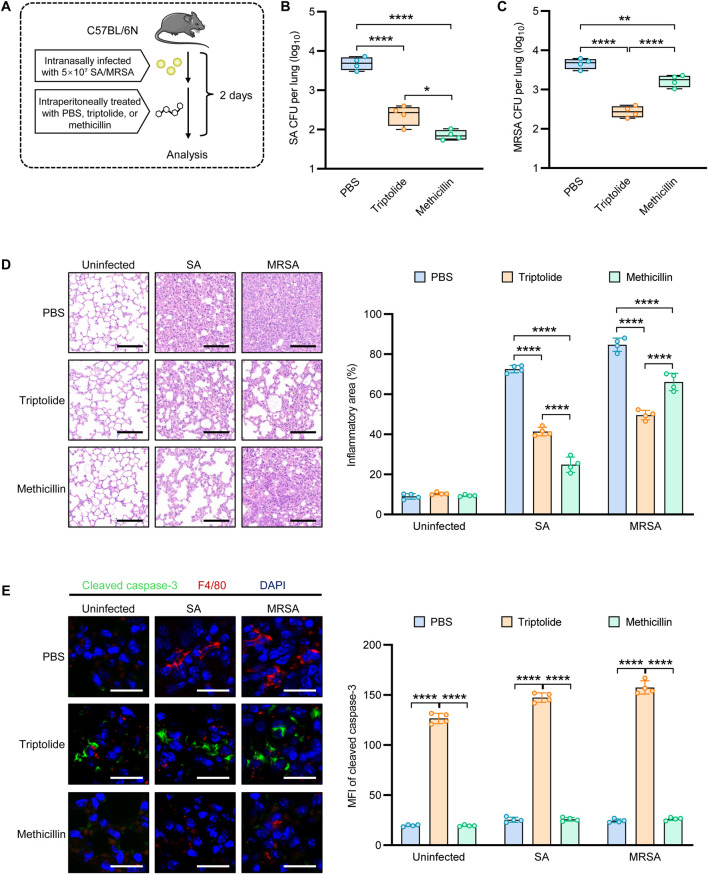
Triptolide reduces SA and MRSA survival and attenuates host inflammatory pathology *in vivo* **(A)** Diagram of the experimental design in mice. Bacterial loads of SA **(B)** and MRSA **(C)** in the lungs of C57BL/6N mice treated with PBS, triptolide, or methicillin. **(D)** Representative images and quantitation for inflammatory areas in the lungs of C57BL/6N mice treated as in **(B,C)**. Scale bar, 100 μm. **(E)** Immunofluorescence and quantitation for cleaved caspase-3 (red) in the lung macrophages (green) of C57BL/6N mice treated as in **(B,C)**. Scale bar, 20 μm. Data are shown as mean ± SEM [n = 4 in **(B–E)**]. *0.01 ≤ *P* < 0.5; **0.001 ≤ *P* < 0.01; ***0.0001 ≤ *P* < 0.001; ****P < 0.0001 [one-way ANOVA with Tukey’s post-hoc test for **(B–E)**].

**FIGURE 5:**
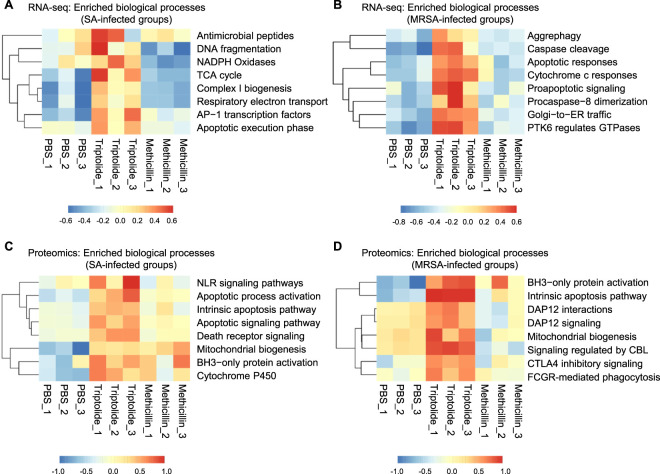
Transcriptomics and proteomics analysis revealing that triptolide induces apoptosis in lung macrophages from mice infected with SA or MRSA. **(A)** GO enrichment analysis of differentially expressed genes in SA-infected mice treated with PBS, triptolide, or methicillin. **(B)** GO enrichment analysis of differentially expressed genes in MRSA-infected mice treated with PBS, triptolide, or methicillin. **(C)** GO enrichment analysis of differentially expressed proteins in mice treated as in **(A)**. **(D)** GO enrichment analysis of differentially expressed proteins in mice treated as in **(B)**.

To evaluate the therapeutic potential of triptolide against SA and MRSA *in vivo*, we established a murine infection model via intranasal inoculation. Infected mice were concurrently treated with PBS, triptolide, or methicillin via intraperitoneal injection (Figure 4A). Through analyzing the pathogen burdens in mouse lungs, we found that triptolide significantly reduced SA loads, with efficacy slightly weaker than that of methicillin (Figure 4B). In MRSA-infected mice, methicillin exhibited only limited activity, whereas triptolide markedly decreased MRSA burdens in the lungs (Figure 4C). Consistently, histopathological analysis demonstrated that triptolide attenuated pulmonary inflammation in both SA- and MRSA-infected mice, whereas methicillin was effective only against SA-induced inflammation (Figure 4D). To further confirm that the *in vivo* efficacy of triptolide against SA and MRSA depends on apoptosis, we assessed caspase activation in lung tissues by immunofluorescence, and found that triptolide increased caspase-3 cleavage in macrophages isolated from infected lungs, whereas methicillin failed to induce such an effect (Figure 4E). Corroborating these findings, transcriptomic and proteomic analyses demonstrated enhanced activation of apoptotic signaling pathways in mouse lung macrophages treated with triptolide, but not with methicillin (Figure 5). Collectively, these data indicate that triptolide potentiates host apoptosis-mediated anti-SA/MRSA immunity *in vivo*.

### 3.5 Triptolide maintains microbiota homeostasis and improves metabolic function in infected mice

**FIGURE 6:**
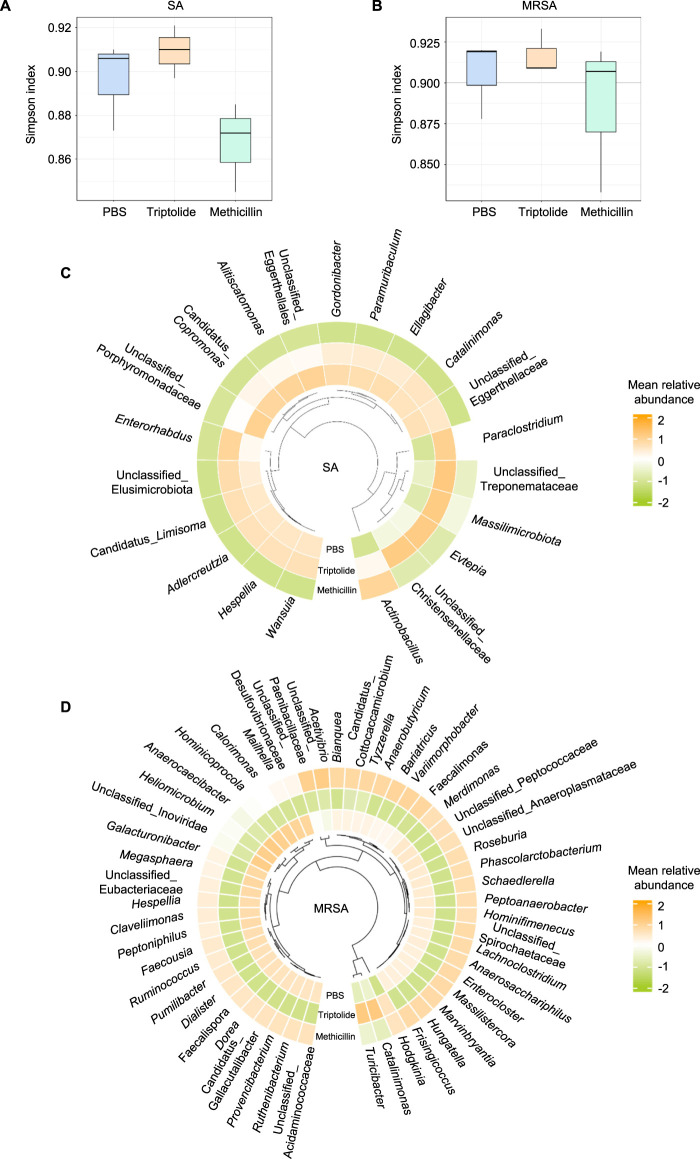
Metagenomics analysis revealing that triptolide maintains microbiota homeostasis in mice infected with SA or MRSA. **(A)** Alpha (α) diversity among SA-infected mice treated with PBS, triptolide, or methicillin. **(B)** Alpha (α) diversity among MRSA-infected mice treated with PBS, triptolide, or methicillin. **(C)** Differential analysis of gut microbial composition among SA-infected mice treated as in **(A)**. **(D)** Differential analysis of gut microbial composition among MRSA-infected mice treated as in **(B)**.

Beyond the serious challenge of antimicrobial resistance, conventional antibiotics can also lead to microbiota dysbiosis, thereby compromising host anti-infection immunity (Fishbein et al., 2023). To explore whether triptolide similarly disrupts microbiota homeostasis, we collected fecal samples from SA- or MRSA-infected mice treated with PBS, triptolide, or methicillin, for subsequent metagenomic analysis. Assessment of alpha-diversity metrics demonstrated that triptolide did not reduce microbial diversity, but rather exhibited a trend toward higher diversity relative to the SA- or MRSA-infected PBS controls (Figures 6A,B). Conversely, methicillin treatment resulted in a significant reduction in alpha-diversity, reflecting a marked loss of community richness (Figures 6A,B). Moreover, differential microbiota analysis revealed that triptolide enriched beneficial genera, including short-chain fatty acid (SCFA) producers such as *Hespellia*, *Ellagibacter*, and *Turiibacter* (Hays et al., 2024; Lagkouvardos et al., 2016; Lynch et al., 2023) (Figures 6C,D), which further produce SCFAs to maintain intestinal barrier integrity, promote immune cell activation, and suppress inflammatory responses (Mann et al., 2024). Conversely, triptolide decreased the abundance of pro-inflammatory or opportunistic pathogens such as *Actinobacillus* and *Hungatella* (Sassu et al., 2018; Cai et al., 2024) (Figures 6C,D). In contrast, methicillin treatment exerted the opposite effect, suppressing the above beneficial genera while facilitating the expansion of opportunistic pathogens (Figures 6C,D). Collectively, triptolide treatment maintains microbiota homeostasis and actively promotes a microbial profile conducive to enhanced host defense.

**FIGURE 7:**
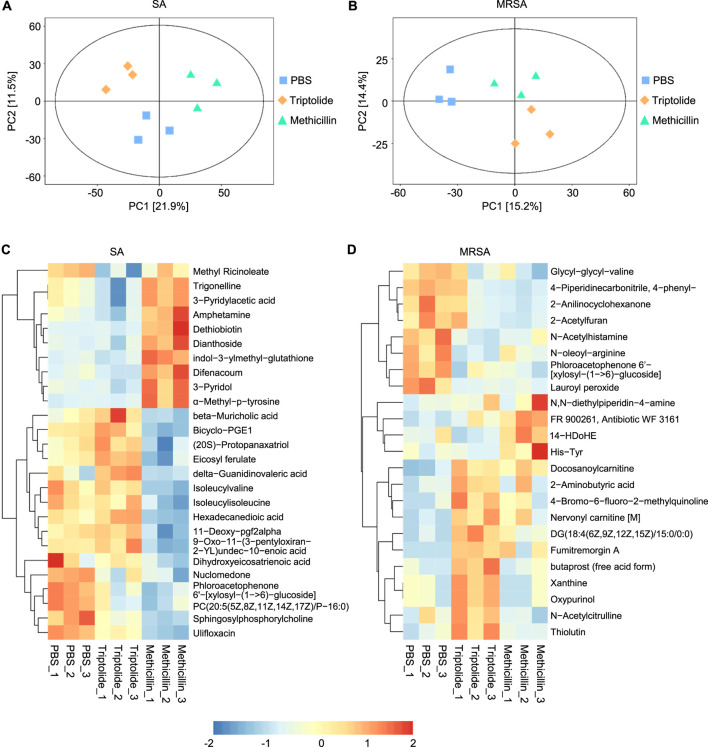
Metabolomics analysis revealing that triptolide enhances immunomodulatory metabolites against infections. **(A)** Partial least-squares discriminant analysis (PLS-DA) score plots of metabolites from SA-infected mice treated with PBS, triptolide, or methicillin. **(B)** PLS-DA score plots of metabolites from MRSA-infected mice treated with PBS, triptolide, or methicillin. **(C)** Heatmap of differential metabolites among SA-infected mice treated as in **(A)**. **(D)** Heatmap of differential metabolites among MRSA-infected mice treated as in **(B)**.

Given that antibiotic-induced gut microbiota dysbiosis can further disrupt host metabolism (He et al., 2023), we thus analyzed the plasma metabolic profiles of SA- or MRSA-infected mice treated with PBS, triptolide, or methicillin by metabolomics. Supervised partial least-squares discriminant analysis (PLS-DA) revealed clear separation among the three groups, indicating distinct metabolic patterns (Figures 7A,B). Differential metabolite analysis further demonstrated that triptolide significantly elevated levels of anti-inflammatory metabolites including (20S)-protopanaxatriol, butaprost (free acid form), and beta-muricholic acid (Jiang et al., 2020; Sharif et al., 2024; Yang and Cong, 2021) (Figures 7C,D). Concurrently, triptolide increased metabolites involved in immune-cell signaling and oxidative-stress homeostasis, such as the second-messenger diacylglycerol DG (18:4/15:0/0:0) and the xanthine oxidase inhibitor oxypurinol (Eichmann and Lass, 2015; Escobar et al., 2012), thereby promoting balanced immune activation (Figures 7C,D). In contrast, these functional metabolites were suppressed in methicillin-treated mice (Figures 7C,D). Collectively, these findings indicate that triptolide treatment improves host metabolic functions to support immune and inflammatory homeostasis. In summary, our study suggests triptolide as a promising lead compound for HDT against SA and MRSA infections by targeting XIAP to induce host apoptosis, while concurrently maintaining microbiota homeostasis and improving metabolic function.

## 4 Discussion

The escalating challenge of SA antibiotic resistance, coupled with its capacity for persistent intracellular infection, urgently demands the development of alternative non-antibiotic therapeutic strategies (Antimicrobial Resistance, 2022; Hommes and Surewaard, 2022). Against this backdrop, HDT has emerged as a pivotal strategy by modulating host immune responses rather than through directly targeting the pathogen (Zheng et al., 2025). Recently, TCM monomers have garnered growing recognition as promising candidates for HDT, owing to their intrinsic multi-target mechanisms, immunomodulatory capabilities, and low tendency to induce antimicrobial resistance (Yang et al., 2021; Liu et al., 2025). However, research on the use of TCM monomers in HDT against SA is limited, particularly regarding their efficacy against intracellular infections. In this study, we identify triptolide as a critical TCM monomer that activates host apoptosis to mediate intracellular clearance of both SA and MRSA, which expands the application of TCM in HDT beyond its established role in inflammation suppression for these infections (Shi et al., 2020; Li et al., 2020; Zhang et al., 2025). Moreover, previous studies on triptolide have predominantly focused on tumors and autoimmune disorders (Bao et al., 2024; Cheng et al., 2021), while its role and mechanisms in infectious diseases remain largely unexplored. Our findings thus advance the understanding of triptolide in this context and establish it as a novel host-directed agent against intracellular SA and MRSA infections. It should be pointed out that we only verified the protective effect of triptolide against SA and MRSA infections over a short 2-day period, without investigating its long-term efficacy, recurrence rate, and safety profile. Although existing studies have reported that the low-dose triptolide used in this study exhibits negligible toxicity during long-term treatment in mice (Xiao et al., 2024; Chen et al., 2024; Kousar et al., 2025), reducing its potential adverse effects during long-term application is still a major challenge for its clinical translation. This challenge could be addressed through the development of triptolide derivatives and novel drug delivery systems (Gao et al., 2021; Song et al., 2023).

Triptolide has long been regarded as a promising pharmacological agent due to its significant anti-inflammatory and immunosuppressive biological activities (Gao et al., 2021). Thus, extensive research has focused on identifying its direct targets to elucidate the underlying mechanisms and facilitate the development of safer, more efficient derivatives. Previous studies have identified several targets of triptolide, including binding to the core subunit XPB of transcription factor IIH and targeting the RPB1 subunit of RNA polymerase II to inhibit gene transcription and DNA repair (He et al., 2015; Kang et al., 2022), targeting peroxiredoxin 2 to induce oxidative stress (Bao et al., 2024), and binding to glucose-regulated protein 78 to interfere with the unfolded protein response and thereby induce endoplasmic reticulum stress (Li et al., 2016). Although these effects can further induce apoptosis, the direct target molecules of triptolide that promote apoptosis, especially the core effector molecules in the apoptotic pathway, remain unknown. Here, we identify that triptolide directly binds to XIAP, the most potent endogenous member of the inhibitor of apoptosis protein family by directly binding to and inhibiting the activities of caspase (Fan et al., 2025). Our data demonstrate that triptolide competitively inhibits the interaction between XIAP and caspases by binding to the BIR2-BIR3 domains of XIAP, which are essential for its caspase-inhibitory activity (Huang et al., 2001). Interestingly, triptolide exhibits a mechanism similar to that of the established XIAP inhibitor SM-164 (Sun et al., 2007), indicating that triptolide may function as a novel, naturally derived XIAP antagonist.

Conventional antibiotic therapies for infectious diseases face challenges not only from drug resistance, but also from the disruption of host microbial ecology due to their broad-spectrum bactericidal properties during treatment (de Nies et al., 2023). Conversely, TCM, with its unique immunomodulatory mechanisms, holds the potential to exert therapeutic effects without compromising microbiota homeostasis (Xue et al., 2023). In our study, compared to methicillin treatment, triptolide demonstrated superior microbiota homeostasis maintenance and increased abundance of beneficial microbes, including SCFA producers such as *Hespellia*, *Ellagibacter*, and *Turiibacter*. The microbiota-modulating effects of triptolide have been previously reported in conditions such as colitis (Wu et al., 2019), diabetes (Lu et al., 2025), and lung injury (Zha et al., 2025), our findings further extend this property by demonstrating that triptolide can also effectively modulate the microbiota in the context of infectious diseases. Furthermore, triptolide increases the levels of metabolites associated with immune activation and anti-inflammatory responses, thereby helping to restore immune and inflammatory homeostasis. It should be mentioned that the specific molecular targets by which triptolide regulates microbiota homeostasis and metabolism remain unclear, and this regulation may be independent of XIAP. Previous studies have shown that triptolide, acting through the bile acid receptor farnesoid X receptor (FXR), modulates the metabolic reprogramming of bile acids, which exert potent selective bactericidal/bacteriostatic effects on gut microbiota (Zha et al., 2025; Li et al., 2025). This suggests that FXR may be targeted (either directly or indirectly) by triptolide to modulate gut microbiota and host metabolism, but this possibility warrants further investigation. Thus, besides targeting XIAP to induce apoptosis for pathogen clearance, triptolide may exert its protective effects against SA infection through multi-target and systemic modulation of the host including gut microbiota homeostasis. Collectively, as a proof-of-principle demonstration, our findings suggest that triptolide represents a promising HDT candidate against SA and MRSA infections, demonstrating a holistic regulatory mechanism that not only enhances pathogen clearance but also maintains host microbiota and metabolic homeostasis. Furthermore, given the broad-spectrum efficacy of HDT strategies, triptolide may offer therapeutic benefits against a wider range of pathogens including complex pathogen co-infections.

## Data Availability

The transcriptomics data presented in the study are deposited in the Gene Expression Omnibus (GEO) database, accession numbers GSE320243 and GSE320244; the proteomics data are deposited in the ProteomeXchange consortium (PRIDE) database, accession numbers PXD074476 and PXD074740; the metagenomics data are deposited in the Sequence Read Archive (SRA) database, accession number SRP676945; the metabolomics data are deposited in the MetaboLights database, accession number MTBLS13930.
