# Supplementary material for: Triptolide clears Staphylococcus aureus infection by targeting XIAP to induce host apoptosis while maintaining gut microbiota homeostasis
Source: Front Pharmacol. 2026 May 26;17:1834558. doi: 10.3389/fphar.2026.1834558 (PMC13246675; doi:10.3389/fphar.2026.1834558)
Supplement: Supplementary file 2 [file Supplementaryfile1.docx]

**Supplementary file**

**Triptolide clears *Staphylococcus aureus* infection by targeting XIAP to induce host apoptosis while maintaining gut microbiota homeostasis**

**Xinli Qiu, Lihua Qiang, Yiru Wang, Bingxi Li, Zehui Lei and Jing Wang**

**The file includes:**

**Supplementary Figures 1–4**


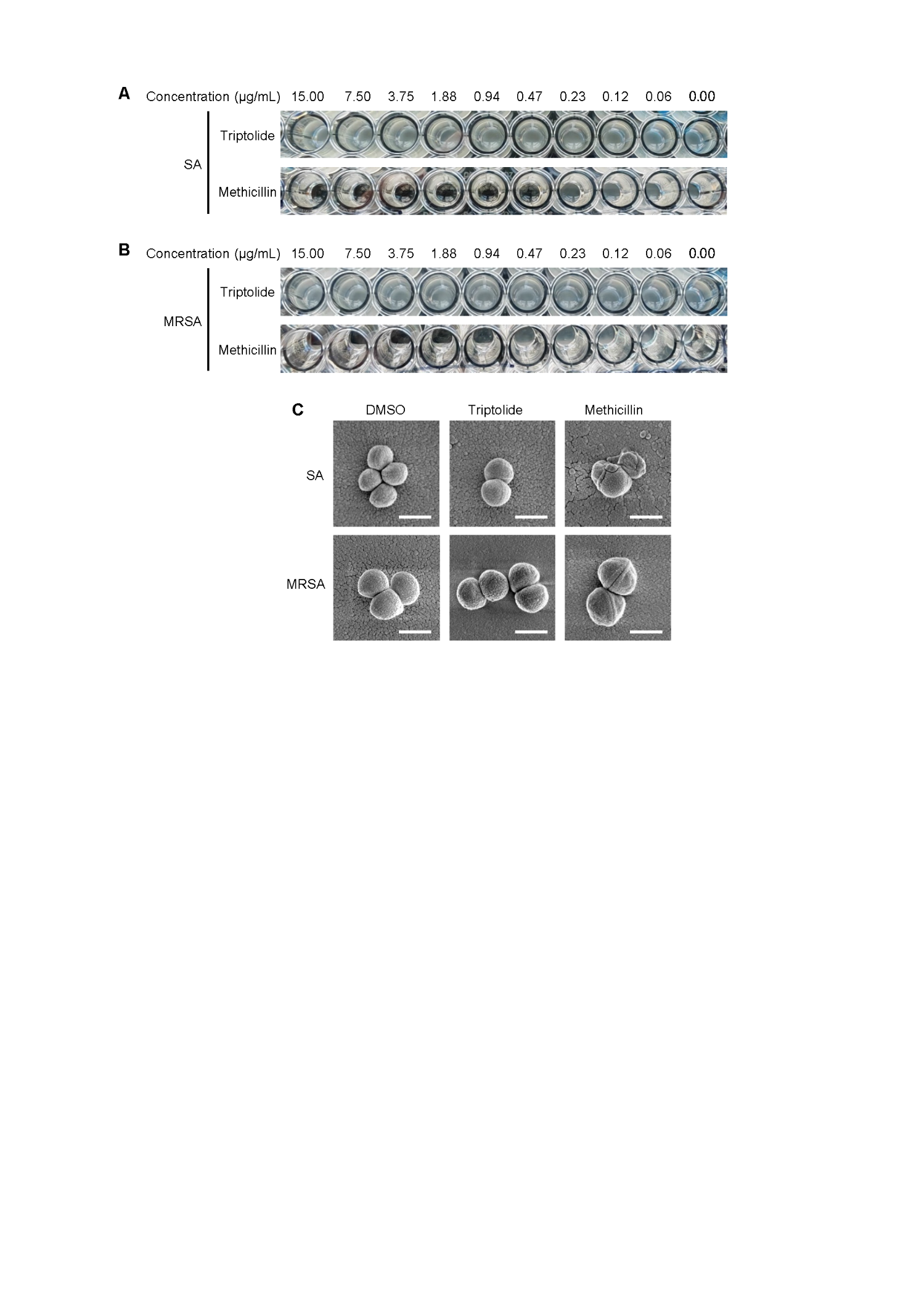


Supplementary Figure S1. Triptolide does not affect the growth and morphology of SA and MRSA. MICs of triptolide against SA **(A)** and MRSA **(B)**. Methicillin was used as the control. **(C)** Representative SEM images of SA and MRSA treated with DMSO, triptolide, or methicillin. Scale bar, 1 μm.


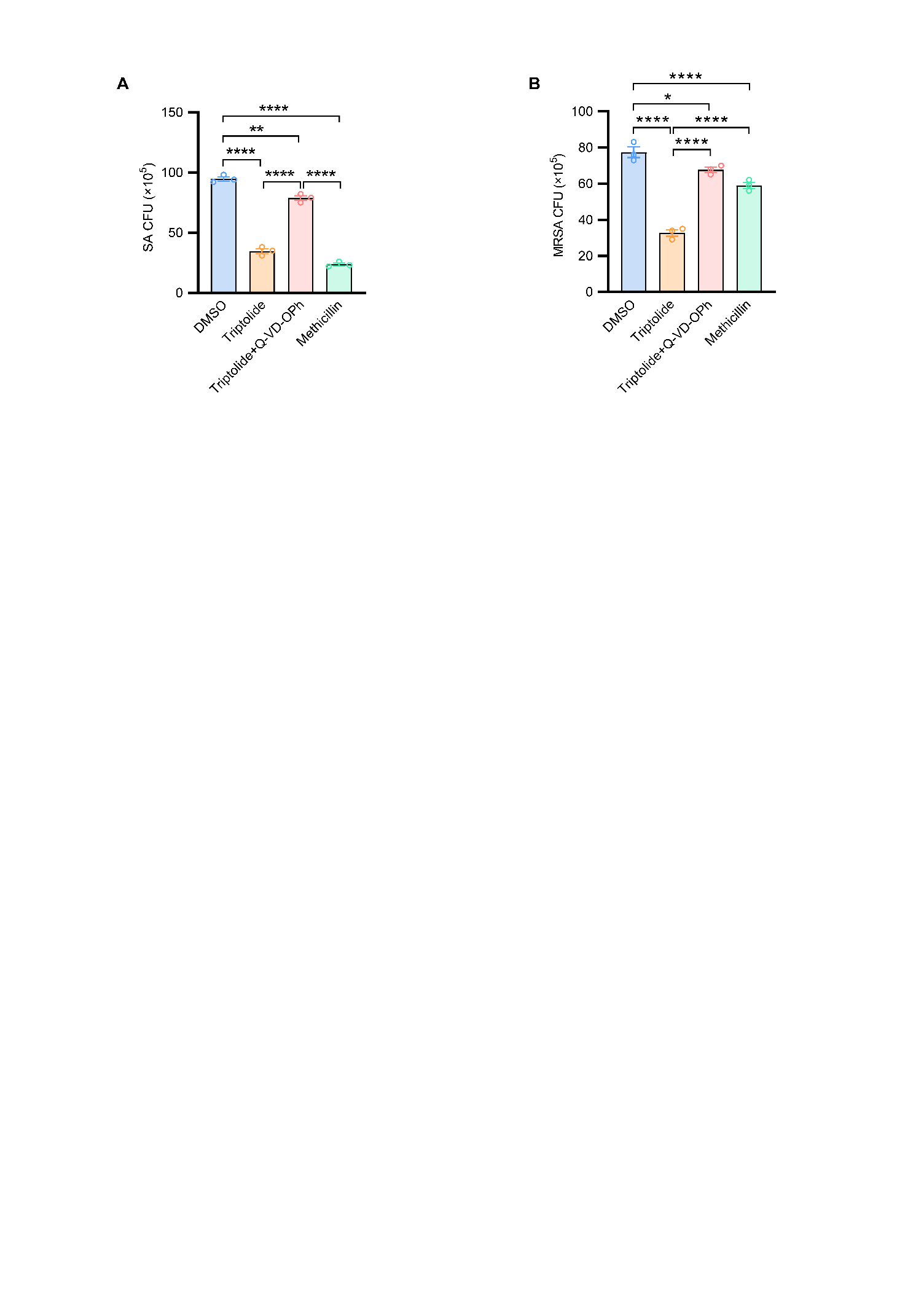


Supplementary Figure S2. Triptolide enhances intracellular clearance of SA and MRSA via apoptosis. Intracellular survival analysis of SA **(A)** and MRSA **(B)** in iBMDMs treated with DMSO, triptolide, triptolide in combination with Q-VD-OPh, or methicillin. Data are shown as mean ± SEM [n = 3 in (A,B)]. **P* < 0.5; ***P* < 0.01; *****P* < 0.0001 [one-way ANOVA with Tukey’s post-hoc test for (A,B)].





Supplementary Figure S3. Molecular dynamics simulation of the interaction between triptolide and XIAP. **(A)** The total number of hydrogen bonds in triptolide-XIAP. RMSF **(B)** and SASA **(C)** curves depicting the interaction between triptolide and XIAP. RMSF, root mean square fluctuation; SASA, solvent-accessible surface area. **(D)** The binding energy of the triptolide-XIAP interaction.


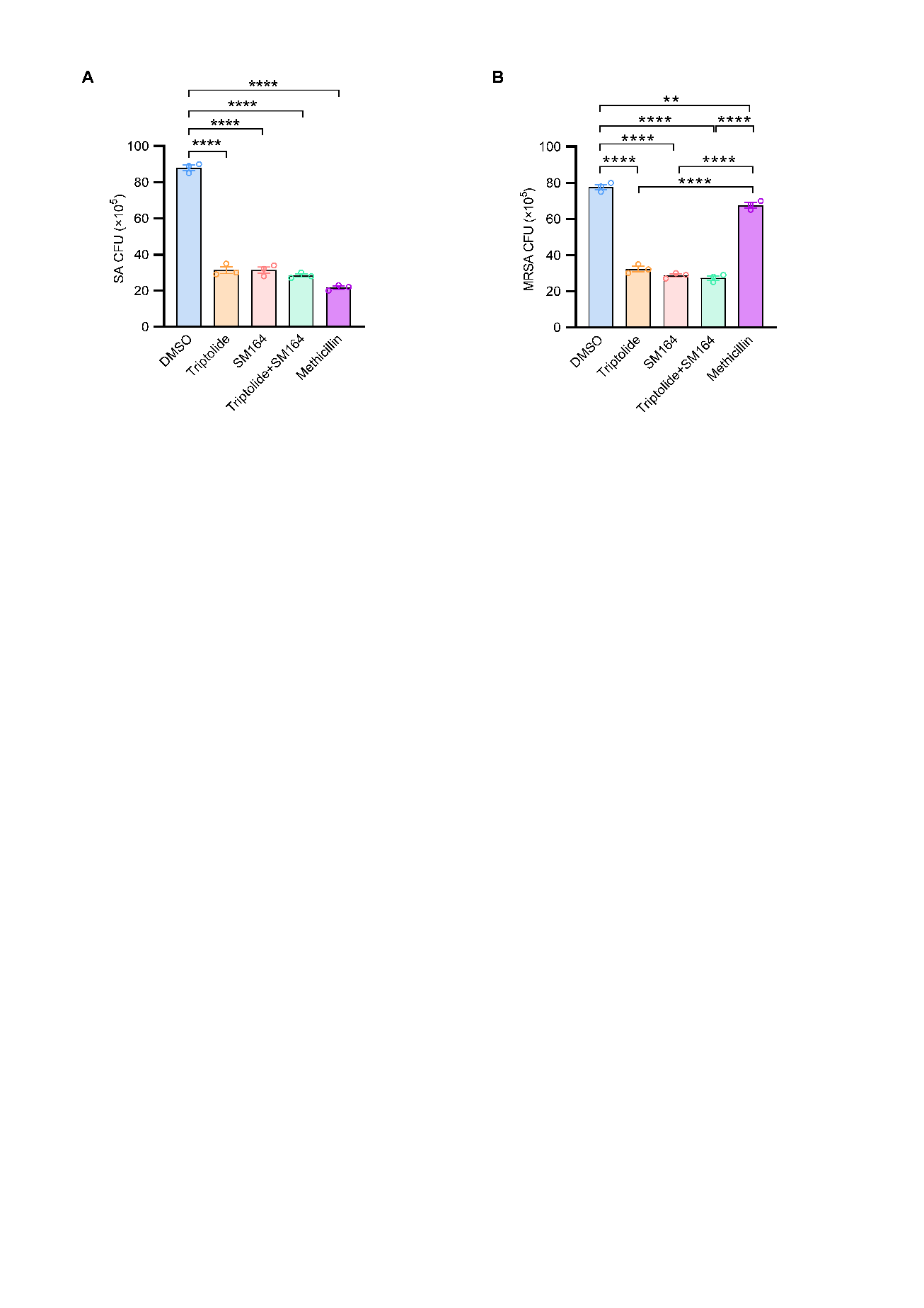


Supplementary Figure S4. Triptolide enhances intracellular clearance of SA and MRSA by targeting XIAP. Intracellular survival analysis of SA **(A)** and MRSA **(B)** in iBMDMs treated with DMSO, triptolide, SM-164, triptolide in combination with SM-164, or methicillin. Data are shown as mean ± SEM [n = 3 in (A,B)]. ***P* < 0.01; *****P* < 0.0001 [one-way ANOVA with Tukey’s post-hoc test for (A,B)].
